# Oral Cholera Vaccination Delivery Cost in Low- and Middle-Income Countries: An Analysis Based on Systematic Review

**DOI:** 10.1371/journal.pntd.0005124

**Published:** 2016-12-08

**Authors:** Vittal Mogasale, Enusa Ramani, Hyeseung Wee, Jerome H. Kim

**Affiliations:** 1 International Vaccine Institute, Policy and Economic Research Department, SNU Research Park, Seoul, South Korea; 2 Korea Development Institute, Sejong-si, South Korea; Massachusetts General Hospital, UNITED STATES

## Abstract

**Background:**

Use of the oral cholera vaccine (OCV) is a vital short-term strategy to control cholera in endemic areas with poor water and sanitation infrastructure. Identifying, estimating, and categorizing the delivery costs of OCV campaigns are useful in analyzing cost-effectiveness, understanding vaccine affordability, and in planning and decision making by program managers and policy makers.

**Objectives:**

To review and re-estimate oral cholera vaccination program costs and propose a new standardized categorization that can help in collation, analysis, and comparison of delivery costs across countries.

**Data sources:**

Peer reviewed publications listed in PubMed database, Google Scholar and World Health Organization (WHO) websites and unpublished data from organizations involved in oral cholera vaccination.

**Study eligibility criteria:**

The publications and reports containing oral cholera vaccination delivery costs, conducted in low- and middle-income countries based on World Bank Classification. Limits are humans and publication date before December 31^st^, 2014.

**Participants:**

No participants are involved, only costs are collected.

**Intervention:**

Oral cholera vaccination and cost estimation.

**Study appraisal and synthesis method:**

A systematic review was conducted using pre-defined inclusion and exclusion criteria. Cost items were categorized into four main cost groups: vaccination program preparation, vaccine administration, adverse events following immunization and vaccine procurement; the first three groups constituting the vaccine delivery costs. The costs were re-estimated in 2014 US dollars (US$) and in international dollar (I$).

**Results:**

Ten studies were identified and included in the analysis. The vaccine delivery costs ranged from US$0.36 to US$ 6.32 (in US$2014) which was equivalent to I$ 0.99 to I$ 16.81 (in I$2014). The vaccine procurement costs ranged from US$ 0.29 to US$ 29.70 (in US$2014), which was equivalent to I$ 0.72 to I$ 78.96 (in I$2014). The delivery costs in routine immunization systems were lowest from US$ 0.36 (in US$2014) equivalent to I$ 0.99 (in I$2014).

**Limitations:**

The reported cost categories are not standardized at collection point and may lead to misclassification. Costs for some OCV campaigns are not available and analysis does not include direct and indirect costs to vaccine recipients.

**Conclusions and implications of key findings:**

Vaccine delivery cost estimation is needed for budgeting and economic analysis of vaccination programs. The cost categorization methodology presented in this study is helpful in collecting OCV delivery costs in a standardized manner, comparing delivery costs, planning vaccination campaigns and informing decision-making.

## Author Summary

We reviewed and re-estimated oral cholera vaccine delivery costs in low and middle income countries standardizing cost categories. The cost categorization proposed here can help in collation, analysis, comparison and economic analysis of OCV delivery costs across countries.

## Introduction

Cholera is transmitted through the fecal-oral route, and humans are the natural host. It is caused by the ingestion of O1 and less commonly O139 serogroups of the *Vibrio cholerae* bacterium and characterized by severe, potentially life-threatening diarrhea [1]. The disease inflicts a significant health burden on many low-and-middle-income countries (LMICs) in settings where food and water are contaminated with human feces. Infrastructure disruption resulting from natural disasters, civil unrest, and war often precipitates cholera outbreaks, particularly in settings where there is endemic cholera risk. Cholera outbreak risk is further increased when infrastructure disruption is superimposed on the poor sanitation and unsafe drinking water found in parts of Africa, Asia, and South and Central America [2]. While improving water and sanitation infrastructure would greatly enhance the control of cholera in the long-term, the use of preventive vaccines has shown promise in the interim [3–5].

The struggle to develop a safe and effective cholera vaccine that can prevent and control the disease has a long history. Injectable whole-cell cholera vaccines were developed as early as the 19^th^ century and extensively used in the 20^th^ century in the Indian subcontinent and later abandoned due to their limited efficacy and systemic adverse events [6,7]. Subsequently, a new generation of live-attenuated or killed oral cholera vaccines were developed, licensed, and deployed. A killed whole-cell cholera vaccine with recombinant B subunit of cholera toxin (Dukoral) was licensed in 1991 (two-dose regimen for >2 years of age) [6] and used by travelers visiting cholera-endemic regions. This vaccine received World Health Organization (WHO) prequalification in 2001 and has a price of $5 per dose on the public market. Meanwhile, Vietnam developed and deployed a locally manufactured OCV, ORC-Vax [8]. The vaccine was licensed in 1997 in Vietnam and was modified to mORC-Vax in 2009 after improving the production process. Currently, the price of this vaccine is US$1.25 per dose on Vietnam’s public market. At the same time, international efforts were made to reformulate ORC-Vax into a less expensive modified killed whole-cell OCV, which was first licensed in India in 2009 (Shanchol, two-dose regimen for >1 year of age), and later WHO-prequalified in 2011. Currently, the price of this vaccine is $1.85 per dose on the public market worldwide. A WHO OCV stockpile was then created in 2013 to make the vaccine available and affordable in emergency settings [9,10]. These two WHO-prequalified OCVs, Dukoral and Shanchol have been deployed in mass vaccination campaigns across many endemic regions either pre-emptively or reactively; notably in Haiti, Comoros, Indonesia, Uganda, Mozambique, Tanzania, India, Bangladesh, Guinea, South Sudan, Malawi, Thailand, Ethiopia and Nepal [11–21].

A cholera vaccination can be broken down into several small and large activities or actions. Understanding the activities involved in vaccination campaigns and estimating cost of each key activity is vital in planning and deployment of OCVs. When deploying a new vaccine, besides routine recurrent costs, the introduction cost such as initial planning, extra logistics and cold chain, training, social mobilization, sensitization, and other new implementation activities such as management of Adverse Events Following Immunization (AEFI) should be considered [22]. Analysis of cost items helps to identify major cost drivers in mass vaccination programs which are critical elements in planning program implementation. This research intends to assess the costs of the different activities required for OCV delivery in LMICs based on systematic literature search and collection of unpublished data from organizations involved in oral cholera vaccination. We propose to categorize cost items in a standardized method and re-estimate delivery costs. Through this analysis we recommend a standardized cost-collation approach for OCV campaigns that can be used in developing OCV delivery cost-estimation tools and comparing costs across different geographical regions.

## Materials and Methods

### Search strategy and selection criteria

A systematic literature review was conducted using search terms (vaccination cost) AND (cholera) in Medline database through PubMed restricting search to humans and dated up to December 31, 2014. Detailed search terms are (("vaccination"[MeSH Terms] OR "vaccination"[All Fields]) AND ("economics"[Subheading] OR "economics"[All Fields] OR "cost"[All Fields] OR "costs and cost analysis"[MeSH Terms] OR ("costs"[All Fields] AND "cost"[All Fields] AND "analysis"[All Fields]) OR "costs and cost analysis"[All Fields])) AND ("cholera"[MeSH Terms] OR "cholera"[All Fields]) AND (("0001/01/01"[PDAT]: "2014/12/31"[PDAT]) AND "humans"[MeSH Terms]). After initial screening on title and abstract, studies using Dukoral, ORC-Vax and Shanchol conducted in LMICs as per the World Bank’s classification [23] that quantified the costing items in cholera vaccination were included. We excluded costing or cost-effectiveness analyses that used simulated or assumed costs, studies that referred to traveler’s vaccination, and studies that considered vaccination in developed countries. The systematic review followed PRISMA guidelines [24] (S1 Checklist). In addition, to find unpublished literature, we searched the Google Scholar and WHO website for OCV mass campaign-related publications and contacted organizations involved in OCV campaigns, including the International Vaccine Institute (IVI), Medecine sans Frontieres (MSF), and the US Centers for Disease Control and Prevention to obtain available reports.

### Categorization of immunization costs

We categorized cost items into four groups with subcategories in each based on the chronological order of implementing OCV campaigns using standardized definitions (Table 1). Vaccination program preparation costs were incurred in field capacity building which includes microplanning, training of personnel, community sensitization, social mobilization and other costs like the storage of vaccines in central warehouses prior to vaccination implementation. Vaccine administration costs included actual vaccine administration costs in the field to individuals as well as transportation of the vaccines from central warehouse to field headquarters and to vaccination field sites. The cost items included are conveyance, per-diem, logistic arrangement, equipment, and location costs for vaccine administration, supervision and monitoring. Finally, all costs related to the AEFI management were included under this category. The last three categories constitute vaccine delivery costs. The vaccine procurement costs included cost of vaccine purchase at preclearance and add-on which comprised costs of freight, insurance, taxes, and customs. This categorization allows comparison of cholera vaccination campaign expenditures across countries that have deployed the vaccines. The financial costs of OCV campaigns were used in our analysis as no opportunity costs were taken into consideration.

**Table 1:** Cost categorization of oral cholera vaccination campaigns.

Main cost category	Sub cost categories	Details of cost items*
1. Vaccination program preparation costs	Micro-planning	Remuneration, travel allowances and per diem for planners, Venue rental (cost) for planning meetings, Computers and office furniture for planning Census update Transportation costs Administration costs Others (write description)
	Training	Remuneration, travel allowances and per diems for trainers and participants Manuals and guides for training Venue rental for training, Transportation costs Administration costs Others (write description)
	Sensitization	Representations/ participation meetings at various levels Others (write description)
	Social mobilization	House-to-house visits Communication through print materials, Radio, television, mobile and internet Others (write description)
	Other preparations	Vaccine handling charges at central store Cold chain equipment purchase or renting Salaries of regional and local vaccination planning management staff Others (write description)
2. Vaccine administration costs	Vaccine logistics and cold chain	Vaccine transport from country headquarters (central warehouse) to field headquarters and to vaccination field sites Staff allowance related vaccine storage Staff allowances related vaccine transport Vaccine storage cost at field headquarters or site Cold chain related costs at field headquarters and site Others (write description)
	Materials and supplies	Water and cups for water Soap for hand wash Incentives to improve active participation Vaccination cards, markers, supplies for data entry, consent form Logistic materials for the site Any other material provided with the purpose of aiding vaccine administration Others (write description)
	Site preparation	Venue rental (cost) for vaccination Cleaning and vaccination booth setup Others (write description)
	Vaccine administration	Salary of staff involved in the vaccine administration,Per diem and other allowances including food and refreshment Labour cost for hired local staff per diem and allowances Transportation costs Administration costs Others (write description)
	Supervision and monitoring	Salary of staff involved in the vaccine administration Per diem and allowances including food and refreshments Labour cost for hired local staff per diem and allowances Review meetings Transportation costs Administration costs Others (write description)
	Waste management	Waste management Others (write description)
3. AEFI management	AEFI monitoring by trained staff	Salary for staff involved in the AEFI management Per diem and allowances including food and refreshments Transportation costs for monitoring Others (write description)
	Medical and advisory service provision	AEFI management related transport costs Health service delivery costs Medicine and laboratory investigations Others (write description)
4. Vaccine procurement	Vaccine price	Vaccine price at pre-clearance Others (write description)
	Shipment related costs	Costs of freight Insurance Taxes Clearance charges Pre-clearance storage cost Transport to central storage Others (write description)

* Costs related to staff from international organizations and research organizations are to be excluded.

### Re-estimation of costs

After categorizing costs from each paper we summarized the results and presented overall vaccination program costs as the sum of all four cost categories. As vaccines are often donated to countries, we differentiated overall vaccination program costs and vaccine delivery costs by segregating vaccine procurement costs. We estimated program cost and vaccine delivery cost per person for complete vaccination using three methods: 1) in United States Dollars (US$) as reported in the literature for the campaign year, 2) in 2014 US$ after adjusting for country level inflation and current exchange rate, and 3) in 2014 international dollars (I$) after adjusting for country level inflation and current purchasing power parity. The year 2014 was selected as the base year for cost analysis.

In Method 1, we presented costs as recorded by the investigators for the campaign year (campaign year cost) in US$. Costs in local currency units (LCU) were converted to US$ based on the World Bank exchange rate reference database for that year [25]. In Method 2, we adjusted the base year costs to 2014 US$ cost-equivalent by first converting the costs to LCU for the vaccination year using the US$-LCU exchange rate for that year and inflating it to year 2014 based on the country inflation rate (inflation, consumer prices, annual %) using the World Bank inflation data [25]. The adjusted results were presented in US$ 2014 after converting LCU to US$ based on the 2014 exchange rate. In Method 3, we adjusted the campaign year cost to the 2014 I$ cost-equivalent by first converting the costs to LCU for the vaccination year, and then inflating to the year 2014 as described for Method 2. The adjusted results were presented in I$ after converting LCU to I$ for 2014 [26].

We employed three methods of program cost estimation for two reasons. First, costs from different campaign years and different countries are not comparable and therefore need to be adjusted to the same base year in order to eliminate inflation effects [27]. Second, the exchange rate conversion does not always consider the differences in the cost of living between countries [28]. For example, the vaccination personnel costs (e.g., per diem) vary by country, which cannot be adequately captured in US$. Purchasing power parity expressed in I$, defined as the number of units of a country’s currency required to buy the same amounts of goods and services in the domestic market as US$ would buy in the United States [28], allows comparison across countries.

## Results

### Systematic review

We identified 83 papers on PubMed search, of which eight were included based on the inclusion-exclusion criteria and two papers were obtained from other sources and personal communications (S1 Flowchart). The program costs for Shanchol delivery were available from four countries (five campaigns) that deployed the vaccine in 2011 and 2014 (Table 2) [15–17,21,29]. A publication presented OCV delivery cost summary for a campaign conducted in three internally displaced people (IDP) camps in 2014 in South Sudan without detailed cost categorization [21]. We also obtained more detailed delivery cost for another OCV campaign conducted in South Sudan in 2013 from personal communications [29]. We were aware that in Ethiopia and Malawi, OCV campaigns were conducted in 2015 and delivery costs were estimated [18], but data was unavailable. The program costs for Dukoral were available from four countries that deployed vaccines from 1997 to 2009 [12–14,20]. The data for Indonesia was obtained from WHO website [20]. In reference review we found one paper presenting a brief description of a Dukoral campaign in Darfur in 2014 stating direct cost of vaccination was US$336,527 or US$ 7 per full vaccinated person [30]. We had to exclude this study from further analysis because costs could not be categorized. The program costs for ORC-Vax were available from Vietnam that deployed vaccine in a 1998 campaign [31].

**Table 2:** Oral cholera vaccination costs as reported, adjusted to US$ 2014 and adjusted to 2014 international dollars.

***Shanchol***	***India*, *2011*** [15]			***Bangladesh*, *2011***[16]^c^			***Guinea*, *2012***[17] ^c^			***South Sudan*, *2013***[29]^c^			***South Sudan, 2014 [21]*** ^c^		
Number fully immunized	23,751			123,661			143,706			71,912			60,421		
Vaccination setting	Preemptive, rural			Preemptive, urban			Reactive, rural			Preemptive, refugee camp, rural			Preemptive, internally displaced people		
Costing perspective	Government			Implementing agency			Implementing agency			Implementing agency			Implementing agency		
	*Method 1*	*Method* 2	*Method* 3	*Method* 1	*Method* 2	*Method* 3	*Method* 1	*Method* 2	*Method* 3	*Method* 1^f^	*Method* 2	*Method* 3	*Method* 1^g^	*Method* 2	*Method* 3
**Currency unit	**US$**	**US$**	Int. $**	**US$**	**US$**	Int. $**	**US$**	**US$**	Int. $**	**US$**	**US$**	Int. $**	**US$**	**US$**	Int. $**
Price per dose	1.00	1.01	3.60	1.00	1.21	3.45	1.00	1.22	2.75	2.40	1.94	9.16	1.85	1.85	7.06
1.Vaccination Program Preparation Cost	5,603	5,655	20,163	17,775	21,465	61,314	4,899	5,995	13,474	103,248	83,568	393,856	NA	NA	NA
1.1 Microplanning	NA	NA	NA	11,915	14,388	41,100	NA	NA	NA	47,250	38,244	180,243	NA	NA	NA
1.2 Training	NA	NA	NA	3,013	3,638	10,393	4,899	5,995	13,474	NA	NA	NA	NA	NA	NA
1.3 Sensitization & Social Mobilization	5,603	5,655	20,163	2,847	3,438	9,822	NA	NA	NA	4,488	3,633	17,120	NA	NA	NA
1.4 Other Preparation Costs	NA	NA	NA	NA	NA	NA	NA	NA	NA	51,510	41,692	196,492	NA	NA	NA
2.Vaccine Administration Cost	17,103	17,261	61,548	183,858^b^	222,029	634,226	268,087^a^	328,079	737,346	168,113	136,069	641,293	104,000	104,000	396,723
3.AEFI Cost	4,237	4,276	15,247	NA	NA	NA	NA	NA	NA	NA	NA	NA	NA	NA	NA
***Total OCV Delivery (Unit) Cost	26,943 (1.13)	27,192 (1.14)	96,958 (4.08)	201,633 (1.63)	243,494 (1.97)	695,540 (5.62)	272,986 (1.90)	334,074 (2.32)	750,820 (5.22)	271,362¶ (3.77)	219,638 (3.05)	1,035,148 (14.39)	104,000 (1.72)	104,000 (1.72)	396,723 (6.57)
4.Vaccine Procurement Cost	122,629	123,762	441,298	284,529	343,600	981,493	642,356	786,102	1,766,734	666,980	539,848	2,544,291	491,048	481,048	1,873,172
Total OCV Program (Unit) Cost	149,572 (6.30)	150,954 (6.36)	538,256 (22.66)	486,162 (3.93)	587,094 (4.75)	1,677,034 (13.56)	915,342 (6.37)	1,120,176 (7.79)	2,517,554 (17.52)	938,342 (13.05)	759,485 (10.56)	3,579,440 (49.78)	595,048 (5.62)	595,048 (5.62)	2,269,896 (21.43)
***Dukoral/ ORC-Vax/mORC-Vax***	***Dukoral*, *Uganda*, *1997***[12] ^d^			***Dukoral*, *Mozambique*, *2003***[13]			***Dukoral*, *Indonesia*, *2005*** [20]			***Dukoral*, *Tanzania*, *2009*** [14]			***ORC-Vax/mORC-Vax, Vietnam, 1997–2012 [31]***		
Number fully immunized	*27*,*607*			*44*,*156*			*54*,*627*			*23*,*921*			*118*,*555*		
Vaccination setting	Preemptive, refugee camp, rural			Preemptive, urban			Preemptive, internally displaced people			Preemptive, urban & rural			Preemptive & reactive, urban & rural		
Costing perspective	Implementing agency			Implementing agency			Implementing agency/government			Government			Government		
**Currency unit**	**US$**	**US$**	Int. $**	**US$**	**US$**	Int. $**	**US$**	**US$**	Int. $**	**US$**	**US$**	Int. $**	**US$**	**US$**	**Int. $** **
Price per dose	*Free*	*Free*	*Free*	*Free*	*Free*	*Free*	4.70	7.23	21.77	*5*.*00*	6.70	17.82	0.31	0.61	1.68
1.Vaccination Program Preparation Cost	NA	NA	NA	11,545	18,650	35,226	8,812	13,550	40,811	21,820	29,251	77,766	4,027	7,953	21,812
1.1 Microplanning	NA	NA	NA	NA	NA	NA	4,423	6,801	20,484	NA	NA	NA	NA	NA	NA
1.2 Training	NA	NA	NA	NA	NA	NA	NA	NA	NA	9,470	12,695	33,751	3,304	6,525	17,896
1.3 Sensitization & Social Mobilization	NA	NA	NA	9,710	15,686	29,627	NA	NA	NA	12,350	16,556	44,015	723	1,428	3,916
1.4 Other Preparation Costs	NA	NA	NA	1,835	2,964	5,599	4,389	6,749	20,327	NA	NA	NA	NA	NA	NA
2.Vaccine Administration Cost	8,634	11,317	28,692	47,998	77,539	146,450	160,360	246,589	742,681	91,000	121,992	324,320	17,720	34,994	95,980
3.AEFI Cost	NA	NA	NA	NA	NA	NA	NA	NA	NA	NA	NA	NA	NA	NA	NA
***Total OCV Delivery (Unit) Cost	8,634 (0.31)	11,317 (0.41)	28,692 (1.04)	59,543 (1.35)	96,189 (2.18)	181,676 (4.11)	169,172¶ (3.10)	260,140 (4.76)	783,492 (14.34)	112,820¶ (4.72)	151,243 (6.32)	402,086 (16.81)	21,747 (0.18)	42,947 (0.36)	117,792 (0.99)
4.Vaccine Procurement Cost	6,021	7,892	20,009	35,136	56,761	107,206	665,247^e^	1,022,967	3,080,981	530,000	710,501	1,888,897	83,700	165,293	453,360
Total OCV Program (Unit) Cost	14,655 (0.53)	19,208 (0.70)	48,701 (1.76)	94,679 (2.14)	152,950 (3.46)	288,882 (6.54)	834,419 (15.27)	1,283,106 (23.49)	3,864,473 (70.74)	642,820 (26.87)	861,744 (36.02)	2,290,983 (95.77)	105,447 (0.89)	208,240 (1.76)	571,153 (4.82)

^¶^ International consultant cost excluded

Note: NA = information not available.

Method 1 reports the survey year costs (US$) presented in the published papers; Method 2 reports the amount after adjusting for country level inflation and converting to 2014 exchange rate (US$); Method 3 after adjusting for inflation and converting to 2014 PPP rate (international dollars).

**Int. $ = International $

***Excludes vaccine purchase cost and other vaccine procurement costs.

South Sudan (EUR 133,916.74 *≈* US$ 182,570), Indonesia (US$ 124,230) and Tanzania (US$ 110,000) had international consultant costs. In Bangladesh, vaccine storage was covered by the EPI free of charge.

^a^ Vaccine administration cost for Guinea also includes parts of the sensitization cost (payments to the sensitization team)

^b^ Bangladesh vaccine administration cost (staff salary) include cost for census update

^c^ Bangladesh (passive surveillance), Guinea and South Sudan monitored adverse event following immunization but did not report associated costs

^d^ Uganda vaccination did not account costs for cold chain (existing cold chain was used at Entebbe, the international arrival point and no cold chain was used at field site), and costs for social mobilization, microplanning and training (carried out by non-governmental organizations). Freight cost includes tax and insurance.

^e^ For Indonesia vaccine cost includes cups for drinking, excludes international experts’ cost (WHO), vaccine administration cost includes staff training and material cost, costing was done from donor’s (WHO) perspective

^f^ Costing for South Sudan was conducted using Euro currency rates. This was converted to US$ rates for February 2, 2013 from oanda.com/currency/converter/: 1Euro to US$ 1.36331

^g^ Costs were not detailed, presented only three cost categories; vaccine price, vaccine shipment and vaccine administration. Total 121,729 doses of Shanchol purchased was not used and the cost is not included in the table.

The number of fully vaccinated people per campaign ranged from 23,751 in India to 143,706 in Guinea. The reported price per dose of Shanchol procurement varied from US$ 1.00 to US$ 2.40 while the price of Dukoral ranged from US$ 0 (free) to US$ 5.00.

### Categorization of costs

The cost categorization and presentation was inconsistent across the studies conducted as shown in Table 2, limiting their comparability. AEFI management, micro-planning, training, sensitization and social mobilization were often mentioned as activities, but costs were merged with other categories which we could not de-merge. In India, micro-planning was considered as a management activity for existing staff and costs were excluded. In Bangladesh and South Sudan, expenses on office furniture and office supplies were categorized under micro-planning while Indonesia had purchased a computer, which was classified as costs for micro-planning. Costs related to AEFI management were reported only from India, representing 15.73% of the total OCV delivery costs.

Of the four major cost categories, vaccine procurement was the costliest component in all OCV campaigns. Whereas, the vaccine administration was the costliest item under vaccine delivery costs (Table 2). Staff salary and allowance when reported, material and supplies such as vaccination card followed by plastic cups, water and soap, when used, were the cost drivers for vaccine administration. Staff salary and allowances are among the costliest items ranging from 23.6% to 87.8% of delivery costs in Guinea and India, respectively. The material and supplies costed 43.9%, 42.1% and 37.0% of delivery costs in Tanzania, South Sudan (2013) and Uganda respectively.

### Re-estimation of costs

**Fig 1:**
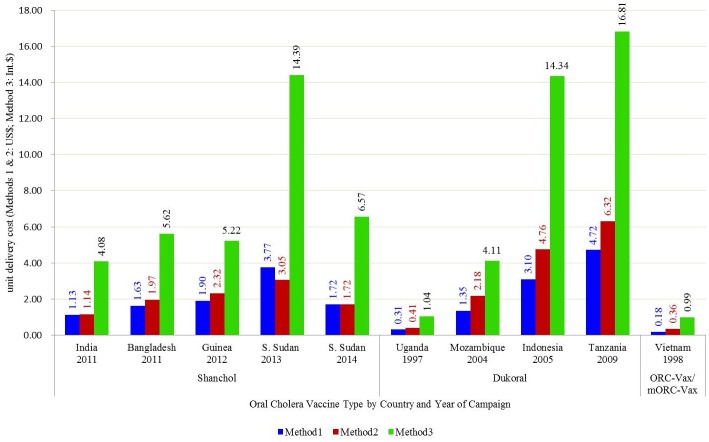
Vaccine delivery cost presented using three different methods (Method 1 = in USD vaccination year, Method 2 = in USD 2014, Method 3 = in I$ 2014).

Costs re-estimated in US$2014 shows high variability of delivery costs across the sites (Fig 1, Table 2). The variability of delivery costs within the same country was also prominent. Although average delivery cost of OCV campaign in South Sudan IDP camps was $1.72 in US$ 2014 (I$6.57) [21], the costs in three different IDP camps were $1.28(I$4.88), $2.02(I$7.71) and $3.38(I$12.89). When prices were adjusted to I$, the costs of vaccination program increased substantially in all settings enhancing the variability across the sites. For example, the cost per unit of OCV delivery increased from US$ 4.70 to I$ 21.77 in Indonesia.

## Discussion

When there is a cholera outbreak or an impending outbreak, there are three main intervention options besides management of cases and public awareness: Do nothing, water and sanitation improvement, and cholera vaccination. The cost of inaction against cholera outbreak could be substantial. One study reported that the drop in exports alone results in substantial trade loss accounting up to 1% of the countries’ GDP [32]. Besides exports, the economic impact of a cholera outbreak includes tourism revenue loss, treatment expenditures and loss of income for those who are affected because they are unable to work. Water and sanitation improvement remains the choice of intervention for cholera and other diarrheal disease control, but requires large investments and takes long-term except personal level interventions such as provision of soap for hand wash and chlorine for water purification. The investments needed for upgrading water and sanitation system is difficult to measure and estimates widely vary. One study estimated that the access to regulated in-house piped water supply with quality monitoring and in-house sewerage connection with partial treatment of sewage for all would require a total investment of US$136.5 billion per year [33]. Oral cholera vaccination is the interim intervention that is effective against cholera, at least in short-term. Accordingly, many OCV campaigns have taken place in different parts of the world, but the costs from those studies have been categorized and presented differently. A well-defined and limited set of basic categories may be more helpful to investigators, health authorities, policy makers, vaccination planners, and community stakeholders. The categories described herein allow for a clear, comparative understanding of vaccination campaign costs that can better guide decision-making.

The delivery costs of OCV through mass campaigns differed by country and even within the same country and same settings. The delivery cost of Shanchol in US$ 2014 varied from $1.14 in India to $3.05 in South Sudan per fully immunized person. The difference was higher in I$2014, ranging from I$4.08 in India to I$14.39 in South Sudan. Some of those differences could be because of the difference in provisions and activities during vaccination as discussed below, while other factors could be that the costs are collected and reported differently. However, the costs of OCV delivery in US$ 2014 in three different IDP settings in South Sudan ranged from $1.28 to $3.38 per fully immunized person. This cost difference could be partially attributed to the scale of vaccination, lowest costs of $1.28 was at IDP camp that vaccinated 38,200 people compared to the highest costs of $3.38 was at IDP camp that covered only 7,400 people. Once cost collection, categorization and presentations are standardized, the costs in US$ should help donors and financing bodies to decide the comparative resources required for vaccination in various settings. Whereas the costs in I$ will be helpful in comparing delivery costs across countries when in-country resources are used partially or completely as the case in many preemptive vaccinations in endemic settings.

Vaccine delivery costs were generally higher for Dukoral than Shanchol, with the exception of Uganda. The higher cost of Dukoral delivery could be partially because of its buffer requirement which complicates vaccine administration process requiring more materials and supplies. A higher proportion of delivery costs are constituted by materials and supply as reported in Tanzania (43.9%), Uganda (37.0%) and Mozambique (28.3%) where Dukoral was used. In Tanzania the material and supply costs were high because it included domestic vaccine storage and transport costs. The delivery costs in Uganda was lower because it did not include costs for program preparation (micro-planning, training, social mobilization & sensitization and other preparation costs) and cold chain costs as the campaign used existing cold chain system for vaccine storage at operational headquarters (Entebbe) and did not use cold chain at field level (Adjumani). Also, vaccination coverage and acceptability survey, and AEFI data collections were not accounted for in the costs. In Uganda, Mozambique and Indonesia the vaccine was air-delivered to the site of vaccination due to difficulty in transport or security reasons or due to the fear of breaking the cold chain, which added substantially to the costs.

The costs of Shanchol delivery were highest in 2013 campaign in South Sudan compared to the other three countries that deployed the vaccine. In South Sudan 2013 campaign, items such as soap, cup and water was provided to Shanchol recipients, resulting in increased proportion of material and supply costs (42.1%). This was higher than other sites such as Bangladesh (13.6%) where these provisions were a part of a research activity and in Guinea (14.3%) where such provisions were part of outbreak preventive measures. Costs for Shanchol delivery were next highest in Guinea because of the transport costs where vaccination teams were mobile on car or boat. The proportion of staff salary and allowances as a part of vaccine administration cost was high in India (87.8%) and Bangladesh (62.7%) compared to Guinea (23.6%) and South Sudan (25.3%). The proportion was high in India because it included staff training costs and in Bangladesh it included costs for pre-vaccination census and intensive house to house mop-up vaccination.

The overall costs for administering ORC-Vax was relatively cheaper in Vietnam compared to other OCVs partly due to the fact that it was integrated into their routine immunization system [31]. This suggests that the routine administration of OCV through existing immunization systems may reduce the vaccine delivery costs. However, the staff costs as a proportion of vaccine administration costs were relatively high (75.9%).

Vaccine procurement accounts for the highest proportion of the total vaccination program costs, the majority of which is due to the cost of the vaccine itself. Even if a country receives donated OCVs, international transportation of the donated vaccines to its borders as well as the clearance of the vaccines at the point of entry accounts for a sizable proportion of the costs. Besides scaling up vaccine supply through the entry of multiple competitive manufacturers [34], a single dose vaccine strategy, if deployed, particularly in outbreak settings is likely to lower vaccine costs [35]. The vaccine administration cost was the next highest because it involves intensive efforts to reach each individual to be vaccinated that needs lot of human resources, cold chain and materials such as vaccination card, soap, water and cups. As Shanchol does not need buffer, administering vaccine without provision of water and keeping OCV outside the cold chain could reduce some of these costs [36].

There are several limitations in our analysis. First, the studies analyzed included only direct costs. The indirect costs—such as loss of income and transportation costs for those who spend time to visit a vaccination post [37] are not accounted for. Adding these vaccine recipient costs would be valuable for a better understanding of the total costs of vaccination and help in developing plans to reduce vaccine recipient’s costs which may improve vaccine acceptance [38]. Two of the Shanchol costing studies later published OCV delivery costs under societal perspective [37,39]. Second, several of the studies included in our analysis organized the costs using their own methods and costing categories, which made it difficult to reorganize the costs for the purposes of our analysis [12–14,20]. This insufficient and unclear information may have resulted in some misclassification of cost categories. Third, we only could include financial costs, not economic costs as most studies presented financial costs. Inclusion of economic costs in future studies is important to understand all the costs-involved in conducting OCV campaigns and also to conduct cost-effectiveness analysis. Fourth, the presentation of costs in selected papers did not allow us to differentiate between fixed and variable costs. The fixed costs will not be affected by a larger OCV introduction while, the variable costs per unit will be further reduced by scaling up the program. It is important to identify and list fixed and variable costs in future costing studies. Fifth, the scope of this work was confined to vaccine delivery costs and it does not include value for money measures such as cost-effectiveness analysis. The reviews on health economic evaluations around OCV delivery will be useful in informed decision making. Finally, availability of unpublished data from two sites (Malawi and Ethiopia) would have improved the cost estimation.

## Conclusion

Understanding the costs of cholera vaccination campaigns is of paramount importance in the economic evaluation as well as in planning future vaccination programs. Currently, there is limited OCV delivery cost data, collected inconsistently and reported capriciously limiting the comparability of costs across settings. Categorizing the costs into easily differentiable categories is useful to the planning process and comparison between campaigns. We recommend that future OCV costing studies include both financial and economic costs and use the cost categories defined in this study for clearer collation, analysis, and comparison of campaign costs.

## Supporting Information

S1 Checklist PRISMA checklist. (DOC)

S1 Flowchart PRISMA flowchart. (TIF)
