# Supplementary figures and images for: Oral Cholera Vaccination Delivery Cost in Low- and Middle-Income Countries: An Analysis Based on Systematic Review
Source: PLoS Negl Trop Dis. 2016 Dec 8;10(12):e0005124. doi: 10.1371/journal.pntd.0005124 (PMC5145138; doi:10.1371/journal.pntd.0005124)

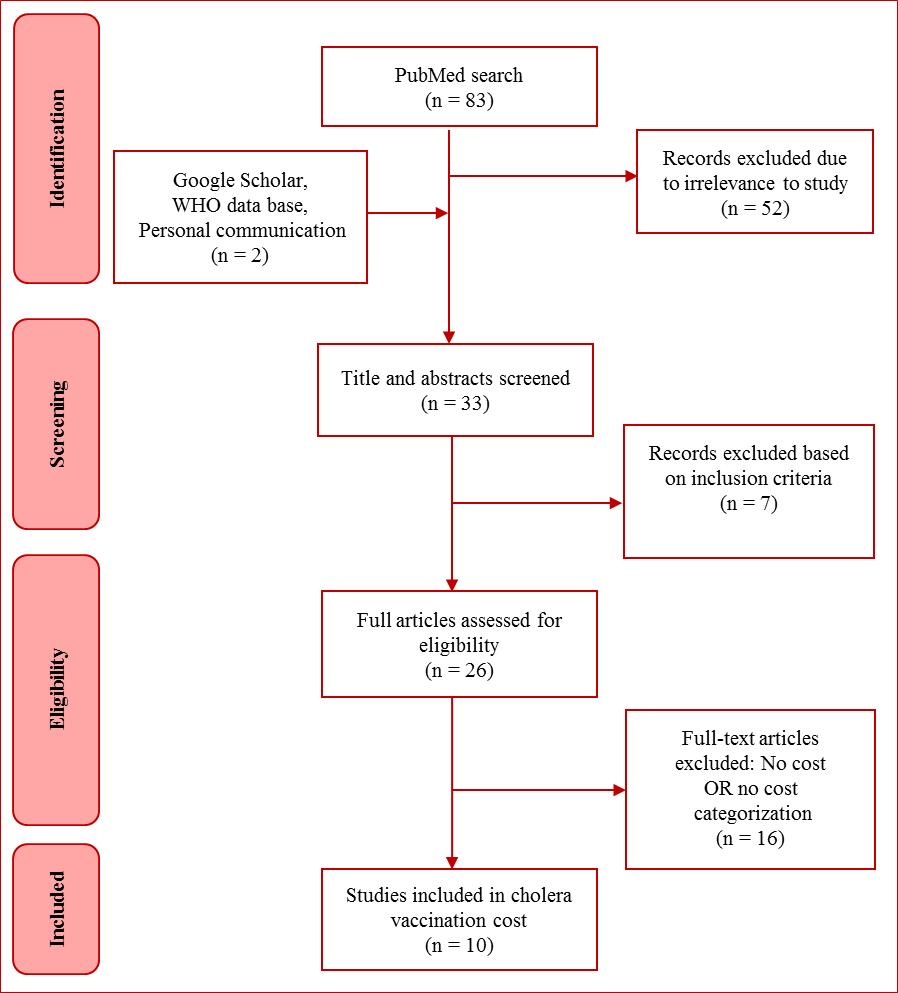

Supplement: S1 Flowchart — (TIF) [file pntd.0005124.s002.tif]
